# Activation of Mast Cells Promote *Plasmodium berghei* ANKA Infection in Murine Model

**DOI:** 10.3389/fcimb.2019.00322

**Published:** 2019-09-10

**Authors:** Bo Huang, Shiguang Huang, Xiaoyan Chen, Xiao Bo Liu, Qiang Wu, Yongfei Wang, Xiaobo Li, Kunning Li, Hongzhi Gao, Shan Cen, Rongtuan Lin, Zhenlong Liu, Xiaobao Jin

**Affiliations:** ^1^Guangdong Provincial Key Laboratory of Pharmaceutical Bioactive Substances, School of Life Sciences and Biopharmaceutics, Guangdong Pharmaceutical University, Guangzhou, China; ^2^Department of Pathogen Biology and Immunology, School of Life Sciences and Biopharmaceutics, Guangdong Pharmaceutical University, Guangzhou, China; ^3^School of Stomatology, Jinan University, Guangzhou, China; ^4^College of Life Science and Technology, Jinan University, Guangzhou, China; ^5^Lady Davis institute for Medical Research, Jewish General Hospital, McGill University, Montreal, QC, Canada; ^6^Division of Experimental Medicine, Department of Medicine, McGill University, Montreal, QC, Canada; ^7^Department of Neurosurgery, The Second Affiliated Hospital of Fujian Medical University, Quanzhou, China; ^8^Department of Immunology, Institute of Medicinal Biotechnology, Chinese Academy of Medical Sciences, Beijing, China

**Keywords:** *Plasmodium berghei*, mast cells, degranulation, pathogenesis, immune responses, vascular permeability

## Abstract

Malaria, a mosquito-borne infectious disease, is a severe health problem worldwide. As reported, some anti-malarial drugs with anti-parasitic properties also block mast cells (MCs) activities. It is hypothesized that MCs activity may be correlated with the pathogenesis of malaria. Thus, the role of MCs on malarial pathogenesis and the involved physiological action and pathways need to be further investigated. This study aimed to investigate the effect of MCs activation on malaria disease severity using KunMing mice with *Plasmodium berghei* ANKA (*Pb*ANKA) infection treated with MCs degranulator (compound 48/80, C48/80) or MCs stabilizer (disodium cromoglycate, DSCG). *Pb*ANKA infection caused a dramatic increase in MCs density and level of MCs degranulation in cervical lymph node (CLN) and skin. Compared with infected control, C48/80 treatment had shortened survival time, increased parasitemia, exacerbated liver inflammation and CLN hyperplasia, accompanied with increase in vascular leakage and leukocyte number. The infected mice with C48/80 treatment also elevated the release of CCL2, CXCL1, and MMP-9 from MCs in CLN and skin, and TNF-α, IFN-γ, CCR2, and CXCR2 mRNA expression in CLN and liver. In contrast, the infected mice treated with DSCG showed longer survival time, lower parasitemia, improved liver inflammation and CLN hyperplasia, followed by a decline of vascular leakage and leukocyte number. Decreased MCs-derived CCL2, CXCL1, and MMP-9 from CLN and skin, mRNA expression in CLN and liver (TNF-α, IFN-γ, CCR2, and CXCR2) were also observed in infected mice with DSCG treatment. Our data indicated that MCs activation may facilitate the pathogenesis of *Pb*ANKA infection.

## Introduction

Malaria is one of the most severe mosquito-borne infectious diseases worldwide. It has caused ~219 million clinical cases and ~435,000 deaths in 2017, and ~90% of malaria cases and deaths were in African regions (WHO, 2018). *Plasmodium* infection is transmitted to humans via the bites of female *Anopheles* mosquitoes carrying parasitic protozoans of the genus *Plasmodium*. *Plasmodium* infection can lead to complex manifestations in patients from asymptomatic, mild (e.g., fever, sweating, and chill), to severe [e.g., anemia, cerebral malaria (CM), pulmonary edema, and acute kidney injury] symptoms. Although more and more evidences have suggested that the clinical manifestations of this disease be primarily based on the delicate balance between pro-inflammatory and anti-inflammatory responses triggered by *Plasmodium* infection (Tannous and Ghanem, 2018), the pathogenesis of this infection still remains incompletely understood.

Mast cells (MCs), effector cell type with dense granules in cytoplasm, are found in all tissues in proximity to blood vessels, nerves, and lymphatic vessels (Gersch et al., 2002). Apart from playing a central role in allergy and anaphylaxis, MCs are currently considered as an important immune effector and modulatory cell upon encounter with incoming different kind of pathogens, including medical protozoan parasites (Cardamone et al., 2016). It was reported that MCs and MCs-derived TNF-α deteriorated pathology and increased parasitic burden in a susceptible strain (BALB/c) of mice during *Leishmania* infection, but improved pathology and decreased parasitic burden in resistant strains (C57BL/6 and CBA/T6T6) of mice (Saha et al., 2004). Similarly, the absence of MCs induced rapid lethality in MC-deficient mice (W/W^v^) orally infected with cysts of the ME49 strain of *Toxoplasma gondii* (*T. gondii*), suggesting that MCs may play a protective role in host defense against oral *T. gondii* infection (Cruz et al., 2014); On the contrary, inhibition of MCs with DSCG drug decreased parasitic burden and extended survival time of KunMing (KM) outbred mice intraperitoneally (i.p.) with the RH strain of *T. gondii* (Huang et al., 2013a). Thus, the accumulating studies have demonstrated that MCs number and level of MCs degranulation have protective and/or pathological impacts on protozoan parasite infections (e.g., *Leishmania* spp., *T. gondii*, and so on) in different settings (Lu and Huang, 2017). It is well-known that *Plasmodium* spp. can infect red blood cells and be in close contact with blood vessels for most of the malaria parasite life cycle in host. Since MCs reside in proximity to blood vessels, instantly release various mediators, and undergo repeated rounds of degranulation and regranulation, it is reasonable to postulate that MCs may mediate the pathogenic process of malaria infection.

The elevated level of IgE antibody, which binds to FcεRI receptors on the surface of MCs, can subsequently induce degranulation and the release of mediators with the aggregation of antigens in experimental and human malaria infection (Duarte et al., 2007; Blank and Mécheri, 2011). Some studies demonstrated that IgE antibody played a protective and/or pathological role in mediating the malaria infection (Duarte et al., 2007; Blank and Mécheri, 2011). On the contrary, other reports showed that MCs-FcεRI receptors for IgE were not involved in the pathogenesis of experimental cerebral malaria (ECM) (Porcherie et al., 2011). It was reported that *P. falciparum* can trigger human skin dermal MCs degranulation, suggesting that the degree of MCs degranulation may be correlated with elevated parasitemia and disease severity (Wilainam et al., 2015). Conversely, it had been demonstrated that MCs-derived TNF had crucial role in host defense against *Plasmodium berghei* ANKA (*Pb*ANKA) infection in murine model (Furuta et al., 2006). Further, MCs activation stimulated by different *Plasmodium* species (*P. chabaudi* AS or *Pb*ANKA) can improved or deteriorate the malaria pathogenesis, respectively (Engwerda and Kumar, 2013; Guermonprez et al., 2013; Theoharides, 2015). These studies indicated that MCs may lead to different outcomes of malaria disease severity possibly due to differences in *Plasmodium* species or selective release of different mediators (Theoharides et al., 2007; Lu and Huang, 2017). Thus, the role of MCs-mediated immune responses in malaria pathogenesis is controversial and remains to be extensively investigated. Currently, the compound 48/80 (C48/80) acts as a “MCs degranulator” as it bypasses IgE-FcεRI mediated MCs degranulation signaling pathway, inhibits calmodulin, and acts directly on G-proteins to stimulate the secretory event. In contrast, disodium cromoglycate (DSCG) serves as a powerful MCs degranulation stabilizer. DSCG restrains the release of allergic mediators from MCs by stabilizing the granule membranes or by blocking calcium channels located on the MCs surface. Thus, C48/80 or DSCG has been widely used to degranulate MCs or prevent MCs degranulation in live animals, respectively. In this study, we infected the lethal murine malaria model of KM outbred mice with *Pb*ANKA to investigate the effects of MCs degranulator (C48/80) and MCs stabilizer (DSCG) on malaria disease severity. Our finding provides insights on MCs activation during *Pb*ANKA infection.

## Methods

### Mice and Ethics Statement

Female KunMing (KM) outbred mice aged 5- to 6- weeks were provided by the Animal Center of Guangdong Pharmaceutical University, PR China. KM mouse is one of the most important strains of mice widely used outbred colony in China. This mouse strain was derived from Swiss mice from the Indian Haffkine Institute in 1944 with the characteristics of high disease resistance, good adaptive capacity, high breeding coefficient and good survival rate (Shang et al., 2009). KM mice with *Pb*ANKA infection have been widely employed to study malaria pathogenesis, and showed high mortality, hyperparasitemia, and pathological lesion in brain, liver or lung tissue) (Huang et al., 2013b; Ding et al., 2016). All mice were housed in pathogen-free environment and received a commercial basal diet and sterile drinking water *ad libitum*. All experimental protocols were reviewed and approved by the Animal Ethics Committee of Guangdong Pharmaceutical University (No. 2012L0816), and deemed to be in accord with the national guidelines for the care and use of animals.

*Pb*ANKA strain was stored at −80°C. Donor female KM mice were inoculated i.p. with frozen stock of *Pb*ANKA-infected red blood cells (iRBCs) and blood smears were examined at 2 days intervals to monitor parasite growth. When the parasitemia reached around 15–20%, KM mice were infected i.p. with 10^6^ iRBCs suspended in 0.2 ml of saline. Two microliter blood of infected mice were collected from the tail vein, and parasitemia was monitored under light microscope ( × 1000) by Giemsa-stained thin blood smears.

### MCs Activation and Stabilization in Murine Malaria Model

To determine whether MCs activation may promote the *Pb*ANKA infection, the experimental procedures of MCs degranulation by Compound 48/80 (C48/80) or stabilization by disodium cromoglycate (DSCG) were carried out as described protocols previously with minor modifications (Huang et al., 2012, 2013a). There were six different groups in this study (e.g., Naive, C48/80, DSCG, Pb, Pb+C48/80, and Pb+DSCG groups). The uninfected mice received daily i.p. injection of saline, C48/80 (Sigma-Aldrich, 4 mg/kg), or DSCG (Sigma-Aldrich, 25 mg/kg) were defined as Naive, C48/80, or DSCG group, respectively. In Pb group, the mice were i.p. infected with 10^6^ *Pb*ANKA-infected red blood cells (iRBCs), and then received daily i.p. injections of saline after infection. Meanwhile, in Pb+C48/80 or Pb+DSCG group, mice subjected to *Pb*ANKA infection received daily i.p. injection of C48/80 (4 mg/kg) or DSCG (25 mg/kg). To avoid animal suffering for a long time, the animals were euthanized if animal suffered certain degree of pain (e.g., inability to eat or drink, or non-responsive to stimuli such as manual prodding) or lost 20% of weight, and then the authors stated that animals died. Tail vein blood was collected from mice of each group, and parasitemia was monitored daily by thin blood smears Giemsa staining. More than 20 fields per smear of mouse (*n* = 6/group) were randomly selected to analyze the percentage of iRBCs with a hematocytometer under a Leica DM 2500B microscope (Leica, Germany) at a magnification of × 1,000. The mice were also monitored daily for survival and symptoms until the termination of the experiment. Infected mice that displayed neurological signs (e.g., ataxia, paralysis, convulsion or coma, loss of reflex, or hemiplegia) and died between days 6 and 8 post-infection (p.i.) were considered suffering from ECM. The experiment was repeated three times and all the analyses were performed by two researchers independently.

### Toluidine Blue Staining for MCs

MCs in cervical lymph node (CLN) or skin tissue was evaluated by toluidine blue staining according to previous report with minor modifications (Huang et al., 2013a). In brief, the tissue (CLN or skin) was immersed in 4% neutral buffered formalin for 48 h before being cut into non-contiguous 5-μm-thick sections (100-μm distance between sections) using a Leica microtome (Leica, Germany). These sections were deparaffinized, rehydrated, immersed in 0.5% toluidine blue (Sigma-Aldrich, USA) for 12 h, and washed in distilled water for 3 min, with three changes. The sections were then dehydrated quickly through 95% ethanol, and two changes of absolute ethanol. The sections were cleared in xylene and mounted with neutral resins. MCs with deep blue-purple staining were analyzed under a Leica DM 2500B microscope. The number of MCs (both intact and degranulated forms) was analyzed by counting three non-contiguous sections (CLN or skin tissue) in each animal (*n* = 6/group). Meanwhile, the area of section was visualized and measured by using Image-pro Plus software in Leica DM 2500B microscope. The density of MCs (both intact and degranulated forms) in CLN or skin tissue was determined as MCs counts/mm^2^, and the level of degranulated MCs was calculated as follows: ▲ = degranulated MCs counts/(both intact and degranulated MCs counts) × 100%.

### Histopathological Analysis

The paraffin-embedded CLN or liver sections (5-μm) were stained with haematoxylin and eosin (H&E) dye, and then analyzed for histopathological changes by two blinded researchers. The inflammatory foci, in three non-contiguous liver sections per animal (*n* = 6/group), was visualized and analyzed under a Leica DM 2500B microscope at a magnification of × 200. The number of inflammatory foci per field was calculated by counting 10 fields of each section (Cavalcanti et al., 2011).

### Evans Blue Dye Perfusion for Vascular Leakage

Vascular leakage in mice was determined using Evans blue dye staining as previously reported with minor modifications (Chen et al., 2018). Briefly, mice (*n* = 4–6/group) were anesthetized by i.p. injection of pentobarbital sodium and then received intravenously injection of Evans blue dye (2% in PBS, 4 ml/kg body weight) for 30 min. The mice were euthanized with by CO_2_ asphyxiation, and the left ventricle was perfused with 10 ml of 4% formaldehyde in PBS for the removal of intravascular-localized dye. After tissue (liver, lung, or brain) was excised and cleaned of connective tissue, Evans blue was extracted by incubation in 50 ml of formamide at 60°C for 24 h. Absorbance of Evans blue in supernatant was read at 620 nm by using a Model Microplate Reader (iMark, Bio-Rad). The tissue was dried at 80°C for 18 h and then weighed. The concentration of Evans blue dye was determined based on the corresponding standard curves and expressed as ng/mg of tissue weight.

### White Blood Cell Count and ELISA Assays

Blood were collected from mice (*n* = 6/group) at the indicated time points, drawn into heparinized tubes, and analyzed total and differential white blood cell counts by using Advia 120 hemocytometer according to the manufacturer's instruction (Siemens, Germany). Meanwhile, Enzyme-linked immunosorbent assay (ELISA) was performed to determine the protein levels of CXCL1 (Gusabio, China), CCL2 (Gusabio, China), MMP-9 (Gusabio, China), IgE (Gusabio, China), AST (Gusabio, China), and ALT (Gusabio, China) in sera from mice (*n* = 6/group) by using commercial kits according to manufacturer's instruction. Briefly, 96-wells ELISA plates were coated with 10 μl of examined sera in 90 μl of sample diluent for 2 h at 37°C, followed by 3X washes with PBS. The plates were incubated with 100 μl of Biotin-antibody buffer for 1 h at 37°C. Plates were rinsed with PBS three time, and developed by incubation with 100 μl of HRP-avidin for 1 h at 37°C. After washing, 90 μl of TBM substrate with H_2_O_2_ (0.75%) was added into these plates for colorimetric development. Thirty minutes later, the reaction was inhabited by incubation with 50 μl of H_2_SO_4_ (2 M). The absorbance was determined by using a Model Microplate Reader (iMark, USA) at 450 nm. The blood concentrations of target proteins were determined based on the corresponding standard curves.

### Quantitative RT-PCR for Chemokine Receptors

Total RNA was extracted from 80 to 100 mg CLN or liver tissue of mice (*n* = 6/group) with TRIzol reagent (TaKaRa, Japan), quantitated by running RNA sample on a 1.0% agarose gel, quantified by measuring the absorbance at 260 and 280 nm with a NanoDrop 2000 spectrophotometer (NanoDrop Technologies, USA), and then reversely transcribed to produce cDNA with PrimeScript 1 st Strand cDNA Synthesis Kit (TaKaRa, Japan). The cDNA was stored at −80°C until use. Real-time PCR was performed using SYBR Green qPCR Master Mix (TaKaRa, Japan) to determine the relative expression of mRNA transcripts of TNF-α, IFN-γ, IL-4, IL-10, CCR2, and CXCR2 in CLN or liver tissue with a Lightcycler® 480 instrument (Roche Diagnostics, Switzerland). The Primer sequences used for Real-time PCR were listed as followed: TNF-α: Forward: 5′ -CACCACCATCAAGGACTCAAAT-3′; Reverse: 5′-CAGGGAAGAATCTGGAAAGGT-3′; IFN-γ: Forward: 5′-TCAAGTGGCATAGATGTGGAAG-3′; Reverse: 5′-TGCTGTTGCTGAAGAAGGTAGT-3′; IL-4: Forward: 5′-GTCATCCTGCTCTTCTTTCTCG-3′; Reverse: 5′-TTGGCACATCCATCTCCGT-3′; IL-10: Forward: 5′-TGCCAAGCCTTATCGGAAAT-3′ and Reverse: 5′-CTCACCCAGGGAATTCAAATG-3′; CCR2: Forward:5′-GTAGGTAACAATACATCCCGTTTGA-3′ and Reverse:5′-AGGCAGTTGCAAAGGTACTG-3′; CXCR2: Forward: 5′-GTAGGTAACAATACATCCCGTTTGA-3′ and Reverse: 5′-AGACAAGGACGACAGCGAAGA-3′; β-actin: Forward: 5′-GTGCTATGTTGCTCTAGACTTCG-3′ and Reverse: 5′-ATGCCACAGGATTCCATACC-3′. The Real-time PCR reactions were performed in a total volume of 10 μl contaning 3.0 μl of dH_2_O, 0.5 μl of each primer (10 pM), 5.0 μl of SYBR® Premix Ex Taq TM (2 × ), and 1.0 μl of preamplified cDNA (100 ng/μl) according to the manufacturer's instructions. The amplification cycling conditions were performed at an initial denaturation at 95°C for 30 s, followed by 43 cycles of 95°C for 5 s and 60°C for 20 s. Relative mRNA levels of target genes were normalized to the housekeeping gene (β-actin), and the results were represented as the fold amplification in comparison with those from uninfected controls.

### Immunofluorescence Double-Staining for MCs Tryptase-CCL2, -CXCL1, or -MMP-9

After deparaffinization and rehydration, endogenous peroxidase activity in sections of CLN or skin tissue in mice (*n* = 6/group) were inhabited by incubation with 3.0 % H_2_O_2_ in dH_2_O for 10 min at room temperature. The sections were incubated with 10% normal goat serum at room temperature for 10 min in order to achieve non-specific binding. Processed sections were then incubated with monoclonal mouse anti-MC tryptase antibody (1:200 dilution; Abcam, USA), and polyclonal rabbit anti-CCL2 antibody (1:200 dilution; Servicebio, China), polyclonal rabbit anti-CXCL1 antibody (1:100 dilution; Boster, China), or polyclonal rabbit anti-MMP-9 antibody (1:200 dilution; Servicebio, China) at 4°C overnight. After being washed with PBS, sections were further stained with Alexa Fluor® 488 Conjugate anti-mouse IgG (1:200 dilution; CST, USA) and Alexa Fluor® 555 anti-rabbit IgG (1:200 dilution; CST, USA) at room temperature for 1 h in darkness, followed by 3X washes with PBS. The sections were mounted using antifade polyvinylpyrrolidone solution (Beyotime, China) in darkness. Positively MCs tryptase would show green fluorescence; in contrast, CXCL1, CCL2, or MMP-9 would represent red fluorescence; Immunofluorescence positive signals would appear as yellow fluorescence once MCs tryptase and CCL2 (or CXCL1, MMP-9) were superimposed in one vision. Images of MCs tryptase-CCL2 (or CXCL1, MMP-9) were captured using Zeiss LSM780 confocal microscope at × 1000 magnifications (Zeiss, Germany). The number of MCs tryptase-CCL2 (or CXCL1, MMP-9) was analyzed by counting three non-contiguous sections (CLN or skin tissue) in each animal (*n* = 6/group). Meanwhile, the area of section was visualized and measured by using Image-pro Plus software in Leica DM 2500B microscope. The density of MCs tryptase-CCL2 (or CXCL1, MMP-9) in CLN or skin tissue was determined as MCs tryptase-CCL2 (or CXCL1, MMP-9) counts per mm^2^.

### Statistical Analysis

Statistical analysis was performed with SPSS (version 13.0) software. The data were reported as mean ± standard error. Statistical analysis of the data was performed by Log-Rank test, a time-series analysis test, Kruskal-Wallis rank sum test, or Student's *t*-test followed by SSR multiple comparisons tests. A value of *P* < 0.05 was considered statistically significant.

## Results

### Increased MCs Number and Level of MCs Degranulation in *Pb*ANKA-Infected Mice

**Figure 1:**
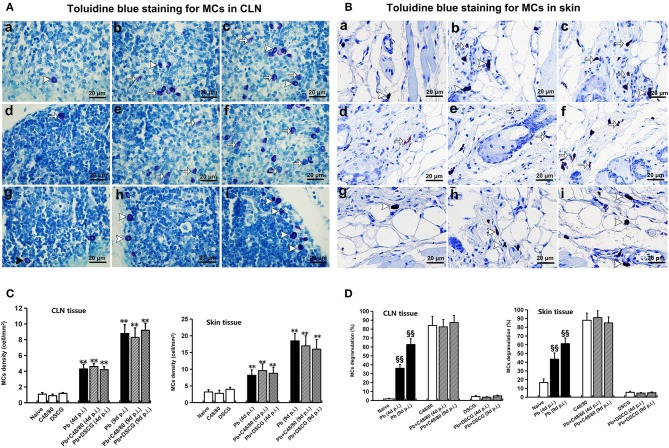
The number of MCs and percentage of MCs degranulation in CLN or skin from PbANKA-infected mice with C48/80 or DSCG treatment. **(A,B)** MCs were evaluated by toluidine blue staining in CLN (magnification, x400) or skin (magnification, x400) from the uninfected mice treated with saline (a), C48/80 (d), or DSCG (g), and the infected mice treated with saline (b), C48/80 (e), or DSCG (h) at 4 days p.i, respectively; infected mice treated with saline (c), C48/80 (f), or DSCG (i) at 9 days p.i., respectively; Intact MCs (arrowheads); Degranulated MCs (arrows). **(C)** The density of MCs in CLN or skin tissue were analyzed from different groups by using Student's *t*-test. *P < 0.05 and **P < 0.01 vs. the Naive mice, ^#^*P* < 0.05 and ^*##*^*P* < 0.01 vs. the infected controls at 4 days p.i., ^&^*P* < 0.05 and ^&&^*P* < 0.01 vs. the infected controls at 9 days p.i.; **(D)** the level of degranulated MCs in CLN or skin tissue were analyzed from different groups by using Student's *t*-test. ^§^*P* < 0.05 and ^§§^*P* < 0.01 vs. the Naive mice, ^*$*^*P* < 0.05 and ^*$$*^*P* < 0.01 vs. the C48/80 group, ^
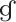
^*P* < 0.05 and ^
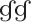
^*P* < 0.01 vs. the DSCG group. There were four to six mice per group, and the data were representative of three experiments.

Toluidine blue-staining showed that the intact MCs were observed in CLN and skin tissues from Naive mice (Figures 1Aa,Ba), and the uninfected/infected mice with DSCG treatment (Figures 1Ag–i,Bg–i); whereas the degranulated MCs were detected in CLN and skin tissues from the infected controls (Figures 1Ab,c,Bb,c), and the uninfected/infected mice with C48/80 treatment (Figures 1Ad–f,Bd–f). As shown in Figure 1C, Naive mice, and the uninfected mice with C48/80 or DSCG treatment exhibited a low density (~1.0 cells per mm^2^) of positively toluidine blue-stained MCs in CLN tissue. *Pb*ANKA infection increased MCs density by ~4.3 fold at 4 days p.i. (4.3 ± 0.6 cells/mm^2^, *P* < 0.01) and by ~8.8 fold at 9 days p.i. (8.8 ± 1.1 cells/mm^2^, *P* < 0.01) compared with those in Naive mice (1.0 ± 0.2 cells/mm^2^). Similarly, in the infected mice treated with C48/80 or DSCG, the density of MCs was notably increased by ~4.6 or ~4.0 fold at 4 days p.i. and by ~9.2 or ~8.3 fold at 9 days p.i. as compared to the uninfected mice treated with C48/80 (0.9 ± 0.2 cells/mm^2^) or DSCG (1.1 ± 0.1 cells/mm^2^), respectively. None of these MCs density showed a significant difference in groups between the infected controls and the infected mice treated with C48/80 (or DSCG) at 4 or 9 days p.i. (*P* > 0.05).

As shown in Figure 1D, the level of degranulated MCs in CLN tissue increased from 1.8 ± 0.6% in Naive mice to 36.1 ± 4.1% (*P* < 0.01) or 62.8 ± 6.5% (*P* < 0.01) in the infected controls at 4 or 9 days p.i., respectively. The administration of C48/80 increased the level of MCs degranulation in CLN tissue in comparison to the infected controls at 4 days p.i. (82.6 ± 8.2% vs. 36.1 ± 4.1%, *P* < 0.01) or 9 days p.i. (87.4 ± 7.8% vs. 62.8 ± 6.5%, *P* < 0.01), respectively. Whereas, DSCG treatment notably reduced the level of MCs degranulation in CLN tissue, compared with those of the infected controls at 4 days p.i. (3.7 ± 0.6% vs. 36.1 ± 4.1%, *P* < 0.01) or 9 days p.i. (5.1 ± 0.9% vs. 62.8 ± 6.5%, *P* < 0.01), respectively. However, no significant difference in the level of degranulated MCs in CLN tissue was found among groups between the uninfected mice treated with C48/80 (84.1 ± 10.1%) and the infected mice treated with C48/80 at 4 days p.i. (82.6 ± 8.2%, *P* > 0.05) or 9 days p.i. (87.4 ± 7.8%, *P* > 0.05). Meanwhile, there was no significant difference in the level of degranulated MCs in groups between the uninfected treated with DSCG (4.5 ± 1.1%) and infected mice treated with DSCG at 4 days p.i. (3.7 ± 0.6%, *P* > 0.05) or 9 days p.i. (5.1 ± 0.9%, *P* > 0.05). Similar patterns of MCs number and level of MCs degranulation were observed in the skin tissue from different groups using toluidine blue staining (Figures 1C,D).

### Parasitemia and Host Survival Modulated by MCs Degranulation

**Figure 2:**
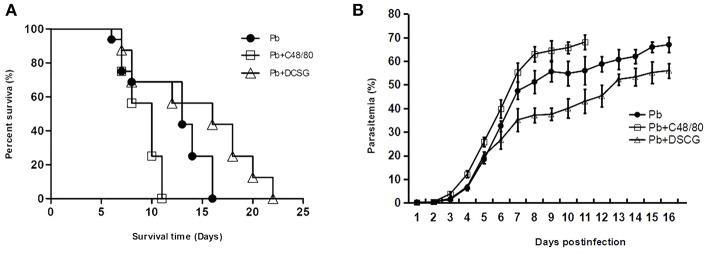
The changes of survival and parasitemia in PbANKA-infected mice with C48/80 or DSCG treatment. Survival **(A)** and parasitemia **(B)** of the infected mice with saline (•, *n* = 16), C48/80 treatment (£, *n* = 16), or DSCG treatment (Δ, *n* = 16) were monitored daily until the termination of the experiment. The experiment was repeated three times with similar results. The significance of differences in survival data or parasitemia data was analyzed by Log-Rank test or a time-series analysis, respectively.

Following infection with 10^6^ iRBCs, the infected control mice (Pb group) died from 6 to 16 days, in which the median survival time for animal was 13 days; whereas the infected mice treated with C48/80 or DSCG died from 7 to 11 days or 7 to 22 days, with the median survival time of 10 or 16 days, respectively (Figure 2A). The administration of C48/80 (10 days, *P* < 0.05) or DSCG (16 days, *P* < 0.05) notably increased or reduced the median survival time compared with those of the infected controls (13 days), respectively. The survival cures among *Pb*ANKA infection with saline, C48/80, and DSCG treatments were significantly different (*P* = 0.0021). Accordingly, the infected mice treated with C48/80 had obvious higher parasitemia than those of the infected controls on days 4–11 p.i. (*P* < 0.05 in all the time points); whereas the parasitemia was markedly lower in the infected mice with DSCG treatment than those of the infected controls on days 6–16 p.i. (*P* < 0.05 in all the time points) (Figure 2B). The ECM mice caused by *Pb*ANKA infection with saline, C48/80 or DSCG treatment were usually moribund from 6 to 8 days. Compared with the infected controls (34.3 ± 3.4%), no significant difference in the incidence of ECM was observed in the infected mice with C48/80 (37.6 ± 1.6%, *P* > 0.05) or DSCG (32.3 ± 2.4%, *P* > 0.05) treatment. The results showed that MC degranulation can affect host parasite growth and host survival.

### Increased CLN and Liver Inflammation by MCs Degranulation

**Figure 3:**
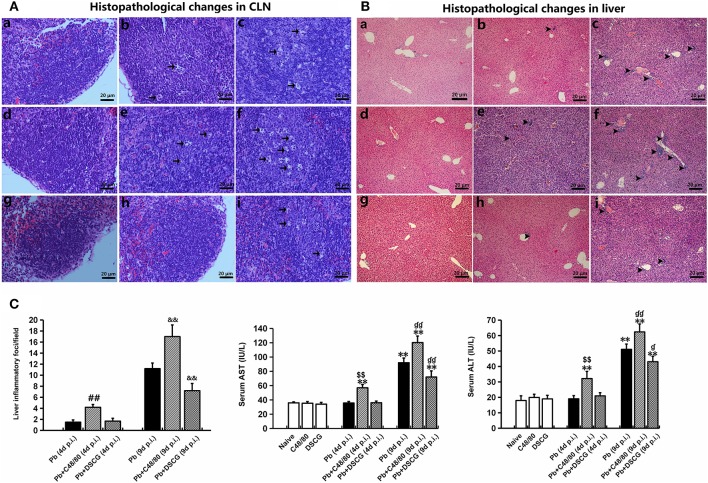
Histopathological examination in CLN or liver tissue from PbANKA-infected mice with C48/80 or DSCG treatment. **(A,B)** Infected mice i.p. inoculated with 10^6^ iRBCs from different groups were killed at 4 and 9 days p.i., and histopathology in CLN or liver was evaluated by H&E staining (magnification, x400). Naive mice (a); infected mice treated with saline at 4 days p.i. (b) and 9 days p.i. (c); uninfected mice treated with C48/80 (d); infected mice with C48/80 treatment at 4 days p.i. (e) and 9 days p.i. (f); uninfected mice treated with DSCG (g); infected mice with DSCG treatment at 4 days p.i. (h); and 9 days p.i. (i); Hyperplasia of germinal center (arrows); Inflammatory foci (arrowheads); **(C)** Liver damage was determined by measuring inflammatory foci counts per field, as well as the protein levels of AST and ALT in sera. There were four mice per group. The experiment was repeated three times, and the data were represented as mean ± SD. Statistically significant differences in liver histopathological score for comparison with the infected controls at 4 days p.i. (^#^*P* < 0.05; and ^*##*^*P* < 0.01) and 9 days p.i. (^&^*P* < 0.05; and ^&&^*P* < 0.01) by using Kruskal-Wallis rank sum test. Statistically significant differences in protein levels of AST or ALT in sera for comparison with Naive mice (*P < 0.05; and **P < 0.01), with the infected controls at 4 days p.i. (^*$*^*P* < 0.05; and ^*$$*^*P* < 0.01) and 9 days p.i. (^
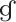
^*P* < 0.05; and ^
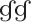
^*P* < 0.01) using Student's *t*-test.

H&E stain showed no hyperplasia of germinal center in lymphatic nodule from the uninfected mice treated with saline (Figure 3Aa), C48/80 (Figure 3Ad), and DSCG (Figure 3Ag), as well as the infected mice treated with DSCG at 4 days p.i. (Figure 3Ah). However, after 4 or 9 days of *Pb*ANKA infection, the enlargement of the follicular germinal centers and hyperplasia were obviously observed in lymphatic nodule from the infected controls (Figure 3Ab,c). Compared with the infected controls at 9 days p.i., the administration of C48/80 resulted in more severe hyperplasia in the infected mice and some cell death (Figure 3Af), whereas the treatment of DSCG led to milder hyperplasia in the infected mice (Figure 3Ai).

No obvious signals of inflammation were found in sections of liver from the uninfected mice treated with saline (Figure 3Ba), C48/80 (Figure 3Bd), or DSCG (Figure 3Bg), as well as the treated with saline (Figure 3Bb) or DSCG (Figure 3Bh) at 4 days p.i. However, severe damage (obvious inflammatory foci) was found in liver tissue of the infected control mice at 9 days p.i. (Figure 3Bc). More severe damage (stronger inflammatory foci) with higher number of inflammatory foci (Figures 3Bf,C) was exhibited in the liver tissues from the infected mice with C48/80 treatment at 9 days p.i. in comparison to the infected controls (17.0 ± 2.1 foci/field vs. 11.2 ± 1.0 foci/field, *P* < 0.01). In contrast, the administration of DSCG attenuated the histopathological damage with a lower number of inflammatory foci in liver tissue from the infected mice (Figures 3Bi,C), as compared to those from the infected controls (7.2 ± 1.3 foci/field vs. 11.2 ± 1.0 foci/field, *P* < 0.01). Liver damage in liver tissue was also determined by measuring the protein levels of aspartate aminotransferase (AST) and alanine aminotransferase (ALT) in sera (Figure 3C). ELISA assay showed that *Pb*ANKA infection dramatically increased the level of AST by 2.6 fold (92.2 ± 6.4 IU/L, *P* < 0.01) and ALT by ~2.8 fold (51.1 ± 3.4 IU/L, *P* < 0.01) at 9 days p.i. related to those in Naive mice (36.0 ± 1.2 IU/L for AST, and 18.0 ± 3.0 IU/L for ALT). Compared with the infected controls at 9 days p.i., there was a significant increase in AST (*P* < 0.01) and ALT (*P* < 0.01) from the infected mice after C48/80 treatment (*P* < 0.01), whereas a significant decrease in AST (*P* < 0.01) and ALT (*P* < 0.05) was recorded in the infected mice after DSCG treatment.

### Increased Vascular Leakage in Liver and Lung by MCs Degranulation

**Figure 4:**
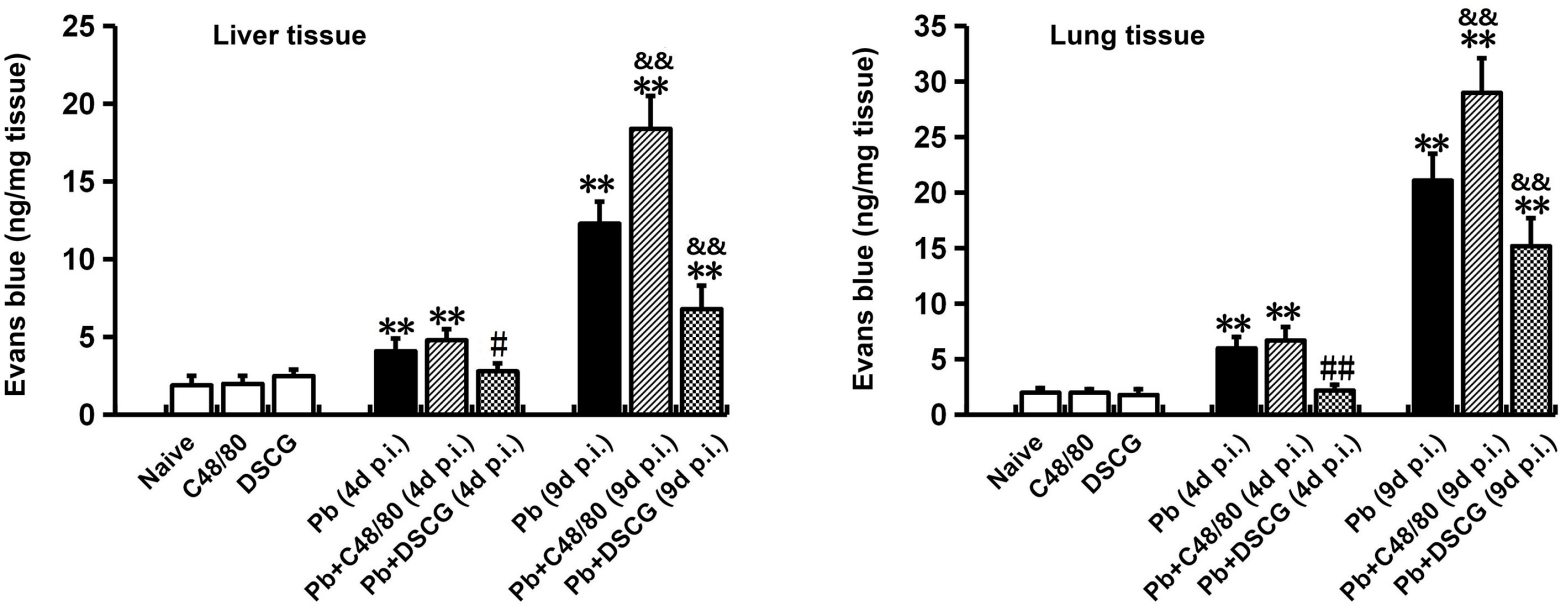
The changes of vascular permeability in liver or lung from PbANKA-infected mice with C48/80 or DSCG treatment. Mice were infected i.p. with 10^6^ iRBCs, and treated with saline, C48/80 or DSCG. At 4 and 9 days p.i., different group of mice (*n* = 4–6/group) were injected 100 μl of 1.0% Evans blue dye via tail veins, and the degree of vascular permeability in liver or lung was expressed as ng/mg of tissue weight. The experiment was repeated three times, and data were presented as means ± SD. *P < 0.05 and **P < 0.01 vs. the Naive mice, ^#^*P* < 0.05 and ^*##*^*P* < 0.01 vs. the infected controls at 4 days p.i., ^&^*P* < 0.05 and ^&&^*P* < 0.01 vs. the infected controls at 9 days p.i. by using Student's *t*-test.

**Figure 5:**
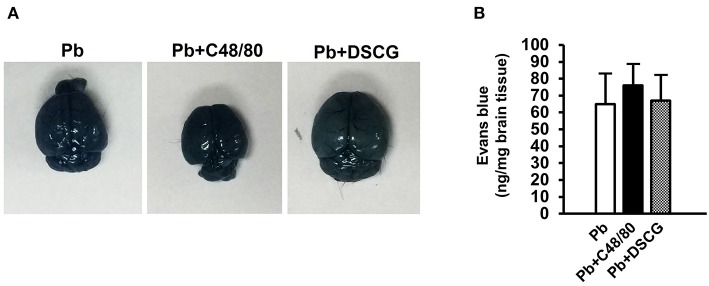
The changes of vascular permeability in brain from ECM mice caused by PbANKA infection with C48/80 or DSCG treatment. ECM mice were randomly selected from PbANKA-infected mice treated with saline (Pb group), C48/80 (Pb+C48/80 group), or DSCG (Pb+DSCG group). Different group of ECM mice (*n* = 4–6/group) were injected 100 μl of 1.0% Evans blue dye via tail veins, **(A)** representative optical image of the brain was captured, and **(B)** the degree of vascular permeability in brain was expressed as ng/mg of tissue weight. The experiment was repeated three times, and data were presented as means ± SD. *P < 0.05 and **P < 0.01 vs. ECM mice in Pb group. The statistical analysis of vascular permeability in brain of ECM mice was performed by Student's *t*-test.

To confirm the role of MCs activation in altering vascular leakage during *Pb*ANKA infection, the leakage of Evans blue dye was measured among different groups (Figure 4). The treatment of C48/80 or DSCG did not notably alter the vascular leakage of liver or lung tissue from Naive mice (*P* > 0.05). *Pb*ANKA infection dramatically increased the vascular leakage of liver tissue at 4 days p.i. by ~2.2 fold (4.1 ± 0.8 ng/mg, *P* < 0.01) and 9 days p.i. by ~6.5 fold (12.3 ± 1.4 ng/mg, *P* < 0.01) related to those in Naive mice (1.9 ± 0.6 ng/mg). In comparison to the infected controls at 9 days p.i. (12.3 ± 1.4 ng/mg), there was a substantial growth (18.4 ± 2.1 ng/mg, *P* < 0.01) in vascular leakage of liver tissue from the infected mice with C48/80 treatment, whereas a major decrease (6.8 ± 1.5 ng/mg, *P* < 0.01) in vascular leakage of liver tissue was observed in the infected mice with DSCG treatment. Similar patterns of vascular leakage expression were observed in the lung tissue. Given the factor that blood brain barrier (BBB) disruption serves as a hallmark of ECM, dye leakage in the brain was examined in ECM mice triggered by *Pb*ANKA infection treated with saline (Pb group), C48/80 (Pb+C48/80 group) or DSCG (Pb+DSCG group). Compared with Pb group (65.4 ± 18.2 ng/mg), no significant difference in dye leakage in the brain was observed in ECM mice selected from Pb+C48/80 group (76.1 ± 12.3 ng/mg, *P* > 0.05) or Pb+DSCG group (67.5 ± 15.2 ng/mg, *P* > 0.05) (Figure 5).

### Increased Leukocyte Number in Blood by MCs Degranulation

**Figure 6:**
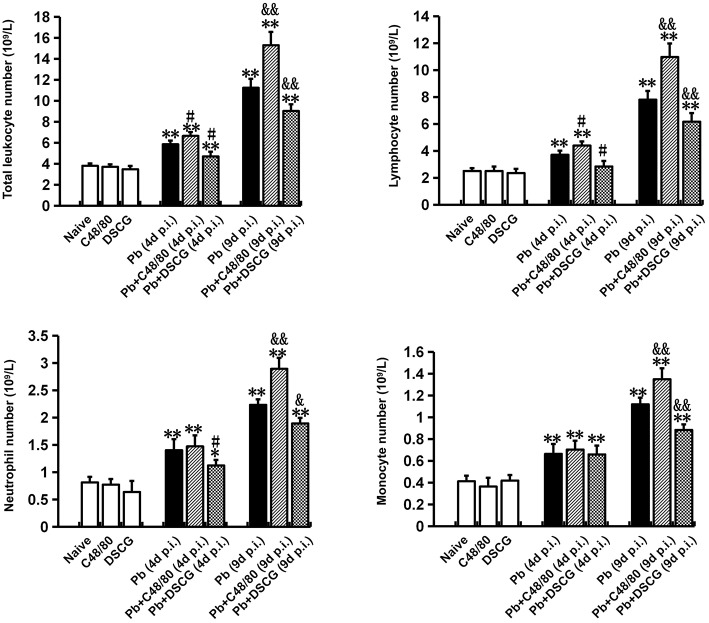
The changes of leukocyte number from PbANKA-infected mice with C48/80 or DSCG treatment. Mice were infected i.p. with 10^6^ iRBCs, and treated with saline, C48/80 or DSCG. At 4 and 9 days p.i., blood from different group of mice (*n* = 4–6/group) was analyzed for the kind and numbers of leukocyte using hemocytometer. The experiment was repeated three times, and data were presented as means ± SD. *P < 0.05 and **P < 0.01 vs. the Naive mice, ^#^*P* < 0.05 and ^*##*^*P* < 0.01 vs. the infected controls at 4 days p.i., ^&^*P* < 0.05 and ^&&^*P* < 0.01 vs. the infected controls at 9 days p.i. by using Student's *t*-test.

To further determine whether MCs activation could alter leukocyte number in blood during *Pb*ANKA infection, full white blood counts in different groups were analyzed (Figure 6). Compared with Naive mice (3.82 ± 0.21 × 10^9^ cells/L), both C48/80 (3.73 ± 0.22 × 10^9^ cells/L, *P* > 0.05) and DSCG (3.49 ± 0.31 × 10^9^ cells/L, *P* > 0.05) treatments did not alter the total number of leukocytes in blood. *Pb*ANKA infection increased the total number of leukocytes by ~1.5 fold (5.87 ± 0.34 cells/L, *P* < 0.01) at 4 days p.i. and by ~2.9 fold (11.26 ± 0.84 cells/L, *P* < 0.01) at 9 days p.i. related to those in Naive mice. Moreover, lymphocytes, monocytes, and neutrophils accounted for the vast majority of leukocyte in the infected controls at 4 or 9 days p.i., while eosinophils, basophils, and plasma cells were rarely observed. In comparison to the infected controls at 4 days p.i. (5.87 ± 0.34 cells/L) or 9 days p.i. (11.26 ± 0.84 cells/L), the total number of leukocytes was risen in the infected mice treated with C48/80 (6.67 ± 0.34 × 10^9^ cells/L for 4 days p.i., *P* < 0.05; 15.31 ± 1.26 × 10^9^ cells/L for 9 days p.i., *P* < 0.01), and declined in the infected mice treated with DSCG (4.72 ± 0.43 × 10^9^ cells/L for 4 days p.i., *P* < 0.05; 9.04 ± 0.63 × 10^9^ cells/L for 9 days p.i., *P* < 0.01). Further, it was shown that the lymphocyte number was predominating among total number of leukocytes at 4 or 9 days p.i. with the frequency of ~63 or ~69%, respectively. Compared with the infected controls at 4 days p.i. (3.72 ± 0.30 cells/L) or 9 days p.i. (7.82 ± 0.64 cells/L), the number of lymphocyte was increased in the infected mice treated with C48/80 (4.41 ± 0.30 × 10^9^ cells/L for 4 days p.i., *P* < 0.05; 10.98 ± 1.00 × 10^9^ cells/L for 9 days p.i., *P* < 0.01), and decreased in the infected mice treated with DSCG (2.86 ± 0.40 × 10^9^ cells/L for 4 days p.i., *P* < 0.05; 6.18 ± 0.63 × 10^9^ cells/L for 9 days p.i., *P* < 0.01). Similar patterns of monocytes and neutrophils expression were found in the blood.

### Increased CCL2, CXCL1, and MMP-9 in Sera by MCs Degranulation

**Figure 7:**
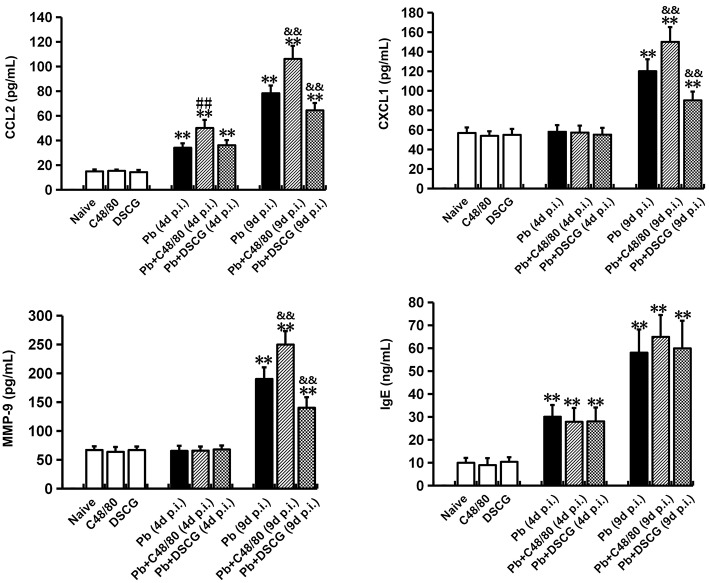
The changes of CCL2, CXCL1, MMP-9, and IgE in sera from PbANKA-infected mice with C48/80 or DSCG treatment. Mice were infected i.p. with 10^6^ iRBCs, and treated with saline, C48/80 or DSCG. At 4 and 9 days p.i., sera were collected from different group of mice (*n* = 4–6/group) and assessed for CCL2, CXCL1, MMP-9, and IgE by ELISA assay. The experiment was repeated three times, and data were presented as means ± SD. *P < 0.05 and **P < 0.01 vs. the Naive mice, ^#^*P* < 0.05 and ^*##*^*P* < 0.01 vs. the infected controls at 4 days p.i., ^&^*P* < 0.05 and ^&&^*P* < 0.01 vs. the infected controls at 9 days p.i. by using Student's *t*-test.

C48/80 or DSCG treatment did not notably alter the protein levels of CCL2, CXCL1, MMP-9, and IgE in sera from the uninfected mice (*P* > 0.05) when compared with Naive mice (Figure 7). However, the higher levels of CCL2 (*P* < 0.01), CXCL1 (*P* < 0.01), MMP-9 (*P* < 0.01), and IgE (*P* < 0.01) were observed from the infected mice at 9 days p.i. than those from Naive mice, respectively. Compared with the infected controls at 9 days p.i., there was a substantial growth in CCL2 (*P* < 0.01), CXCL1 (*P* < 0.01), and MMP-9 (*P* < 0.01) from the infected mice after C48/80 treatment, whereas a major decline in CCL2 (*P* < 0.01), CXCL1 (*P* < 0.01), and MMP-9 (*P* < 0.01) was recorded in the infected mice after DSCG treatment. However, none of the protein levels of IgE showed a statistically significant difference in groups between the infected controls and the infected mice treated with C48/80 (or DSCG) at 4 or 9 days p.i. (*P* > 0.05).

### Increased Cytokine Responses in CLN and Liver Tissues by MCs Degranulation

**Figure 8:**
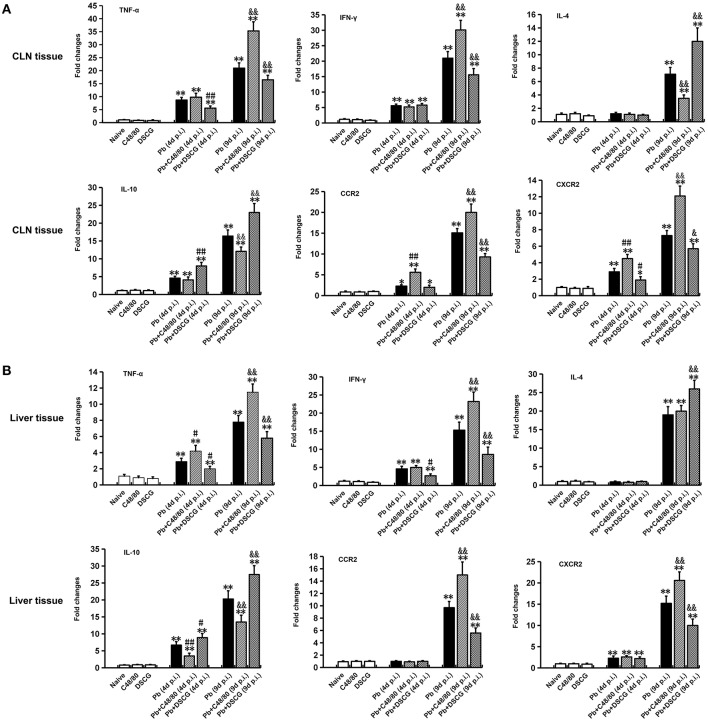
Cytokine and chemokine receptor mRNA expressions in CLN or skin from PbANKA-infected mice with C48/80 or DSCG treatment using qPCR method. **(A)** mRNA levels of TNF-α, IFN-γ, IL-4, IL-10, CCR2, and CXCR2 in CLN tissue; **(B)** mRNA levels of TNF-α, IFN-γ, IL-4, IL-10, CCR2, and CXCR2 in skin tissue. There were 4 to 6 mice per group. The data were representative of three experiments, and presented as means ± SD. *P < 0.05 and **P < 0.01 vs. the Naive mice, ^#^*P* < 0.05 and ^*##*^*P* < 0.01 vs. the infected controls at 4 days p.i., ^&^*P* < 0.05 and ^&&^*P* < 0.01 vs. the infected controls at 9 days p.i. by using Student's *t*-test.

There was no significant difference in all the evaluated cytokines (TNF-α, IFN-γ, IL-4, and IL-10) or chemokine receptors (CCR2 and CXCR2) mRNA expressions of CLN (Figure 8A) or liver (Figure 8B) tissue from uninfected mice with saline, C48/80 or DSCG treatment (*P* > 0.05). The higher level of all cytokines or chemokine receptors examined was observed in the infected mice at 9 days p.i. (*P* < 0.01) in comparison to Naive mice. Compared with the infected controls at 9 days p.i., the C48/80 treatment resulted in a significant increase in the mRNA levels of TNF-α (*P* < 0.01), IFN-γ (*P* < 0.01), CCR2 (*P* < 0.01), and CXCR2 (*P* < 0.01) in the CLN tissue, whereas significantly reduced the mRNA levels of IL4 (*P* < 0.01) and IL10 (*P* < 0.01) in the CLN tissue. On the contrary, the mRNA levels of IL4 (*P* < 0.01) and IL10 (*P* < 0.01) raised, however, compared to the infected controls at 9 days p.i., the mRNA levels of TNF-α (*P* < 0.01), IFN-γ (*P* < 0.01), CCR2 (*P* < 0.01), and CXCR2 (*P* < 0.05) in CLN tissue from the infected mice with DSCG treatment declined. Similar results of cytokines and chemokine receptors mRNA expression were observed in the liver tissue (Figure 8B).

### Increased MCs-Derived CCL2, CXCL1, or MMP-9 in CLN and Skin Tissues by MCs Degranulation

**Figure 9:**
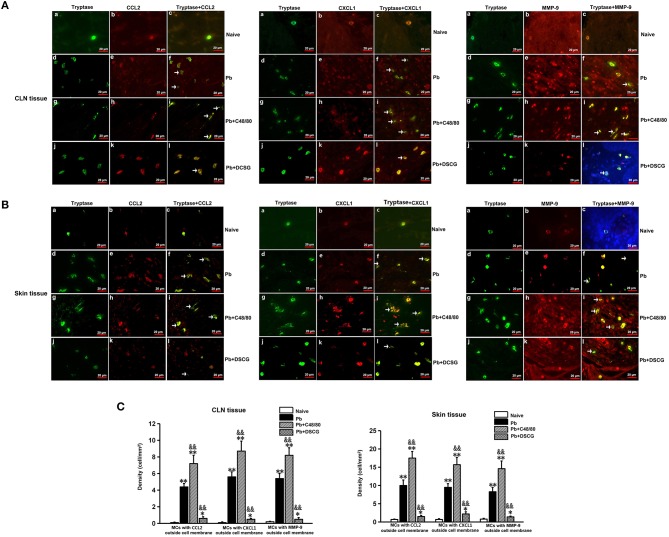
The density of MCs tryptase-CCL2, -CXCL1, and -MMP-9 in CLN or skin from PbANKA-infected mice with C48/80 or DSCG treatment by immunofluorescence staining. **(A,B)** MCs tryptase-CCL2, -CXCL1, and -MMP-9 were evaluated in CLN or skin from uninfected control mice (Naive group), infected mice treated with saline (Pb group), C48/80 (Pb+C48/80 group), or DSCG (Pb+DSCG group) at 9 days p.i. Immunofluorescence positive signals would appear as yellow fluorescence once MCs tryptase and CCL2 (or CXCL1, MMP-9) were superimposed in one vision (magnification, x400). MCs were seen to release CCL2, CXCL1, or MMP-9 when CCL2, CXCL1, or MMP-9 was found outside the MCs cell membrane (arrows). **(C,D)** The density of MCs with CCL2, -CXCL1, or -MMP-9 outside cell membrane in CLN or skin tissue were analyzed from the uninfected control mice, infected mice treated with C48/80 or DSCG at 9 days p.i. The experiment was repeated three times, and data were presented as means ± SD. *P < 0.05 and **P < 0.01 vs. the Naive mice, ^&^*P* < 0.05 and ^&&^*P* < 0.01 vs. the infected controls at 9 days p.i. by using Student's *t*-test.

As shown in Figure 9, MCs were seen to release CCL2, CXCL1, or MMP-9 during *Pb*ANKA infection when CCL2, CXCL1, or MMP-9 was found outside the MCs cell membrane. Semi-quantitative analysis of MCs tryptase-CCL2, -CXCL1, or -MMP-9 in the CLN tissue showed that *Pb*ANKA infection increased the density of MCs with CCL2 (4.40 ± 0.40 cells/mm^2^ vs. 0.10 ± 0.05 cells/mm^2^, *P* < 0.01), CXCL1 (5.60 ± 0.60 cells/mm^2^ vs. 0.10 ± 0.08 cells/mm^2^, *P* < 0.01), or MMP-9 (5.4 ± 0.6 cells/mm^2^ vs. 0.2 ± 0.05 cells/mm^2^, *P* < 0.01) outside cell membrane at 9 days p.i. relative to those from Naive mice, respectively. However, in comparison to the infected control mice at 9 days p.i., the density of MCs with CCL2, -CXCL1, or -MMP-9 outside cell membrane was higher in CLN tissue from the infected mice with C48/80 treatment (*P* < 0.01), whereas lower after DSCG treatment (*P* < 0.01). Similar patterns of the density of MCs with CCL2, CXCL1, or MMP-9 outside cell membrane were detected in the skin tissue. The results show that MCs in CLN (or skin) tissue can release more or lesser CCL2/CXCL1/MMP-9 triggered by C48/80 or DSCG treatment during *Pb*ANKA infection, respectively.

## Discussions

The accumulating studies had indicated that the anti-malarial drugs (e.g., artemisinin derivatives, mefloquine, and ketotifen) not only killed malaria parasite but also blocked MCs degranulation (Milner et al., 2012; Cheng et al., 2013; Paivandy et al., 2014). Moreover, rosiglitazone, which had the ability to inhibit MCs migration and adhesion, extended the survival time and alleviated neurocognitive impairment in ECM (Serghides et al., 2014). It seems reasonable that MCs inhibition may serve as an adjuvant treatment of malaria infection. However, the role of MCs on pathogenesis of malaria needs to be further investigated in order to determine the physiological action and pathways involved. In the present study, our results demonstrated that *Pb*ANKA infection increased the MCs density as well as the percentage of MCs degranulation in the CLN and skin tissues when compared with Naive mice. To further determine whether MCs degranulation can promote the *Pb*ANKA infection in murine model, the *Pb*ANKA-infected KM mice were treated with MCs degranulator (C48/80) and MCs stabilizer (DSCG). Compared with infected controls, the higher level of MCs degranulation was observed in the infected mice treated with C48/80 at 4 or 9 days p.i., accompanied with shorter mice survival time, higher parasitemia, more severe pathology in liver and hyperplasia in CLN; whereas the lower level of MCs degranulation was found in the infected mice with DSCG treatment at 4 or 9 days p.i., followed by longer mice survival time, lower parasitemia, attenuated pathology damage in liver and less hyperplasia in CLN. Our observation is consistent with those in previous reports (Wilainam et al., 2015), where the elevated degree of human skin dermal MCs number and degranulation was found to correlate with increased *P. falciparum* parasitemia and disease severity. Therefore, our data suggest that activated MCs promote pathogenesis of *Pb*ANKA infection and inhibition of MCs improve pathological process.

The data in the present study showed that approximately 30% of the KM mice with *Pb*ANKA infection developed to ECM. Our data is consistent with our early report (Huang et al., 2013b), but is contrast to the other report that 100% of *Pb*ANKA-infected KM mice succumbed to ECM (Ding et al., 2016). It remains an open question that the obvious different incidence of ECM was observed in the same lethal murine malaria model of KM mice infected with *Pb*ANKA. Remarkably, the data presented in this study showed that treatment with C48/80 or DSCG did not promote or prevent the incidence and BBB disruption of ECM, because there should be not enough C48/80 or DSCG drug via i.p. injection cross BBB into brain parenchyma where MCs modulated function and behavior of brain (Hendriksen et al., 2017; Traina, 2017), or KM outbred mice infected with *Pb*ANKA parasite should not be used as an ideal and exclusive ECM model. Thus, further research is needed to fully understand the effect of brain MCs on pathological process of ECM by using an exclusive ECM murine model (e.g., C57BL/6 mice but not KM mice infected with *Pb*ANKA). The controversial role of IgE in regulating the malaria disease severity was demonstrated by a series of studies (Blank and Mécheri, 2011). It was reported that the increase of IgE level was observed in patients with severe malaria in comparison to those with uncomplicated malaria (Perlmann et al., 1994); whereas other studies demonstrated elevated levels of malaria-specific IgE had crucial role in host defense against severe malaria (Bereczky et al., 2004). In the present study, although the elevated levels of IgE in sera were observed *Pb*ANKA-infected mice in comparison to Naive mice, C48/80 or DSCG treatment did not affect the levels of IgE from the infected control mice. Therefore, our data suggested that the aggravated or attenuated pathology damage in the infected mice induced by C48/80 or DSCG treatment may not mainly depend on the level of IgE.

The clinical manifestations of malaria infection mainly depended on a delicate balance between pro-inflammatory and anti-inflammatory responses (Riley et al., 2006). MCs activation can produce and release massive mediators (e.g., cytokines, chemokines, proteases, histamine, growth factors, and so no) that contributes to the modulation of innate and adaptive immune responses (Cardamone et al., 2016). To investigate the effect of mediators released by MCs degranulation on cytokine responses in *Pb*ANKA infection, the mRNA expressions of cytokines (TNF-α, IFN-γ, IL-4, and IL-10) were measured in CLN and liver tissues from different groups. In the present study, the mRNA expressions of TNF-α and IFN-γ in both CLN and liver tissues were notably risen in the infected mice with C48/80 treatment at 9 days p.i. compared with the infected controls; whereas the levels of TNF-α and IFN-γ in both examined tissues were decreased in the infected mice treated with DSCG at 9 days p.i. It was reported that IFN-γ acted as a central cytokine protected against pre-erythrocytic and blood-stage *Plasmodium* parasites (McCall and Sauerwein, 2010), and TNF-α played a crucial role in promoting malaria parasite killing (Randall and Engwerda, 2010). However, overproduction of pro-inflammatory cytokines (e.g., IFN-γ and TNF-α) resulted in the development of severe malaria disease (Perez-Mazliah and Langhorne, 2015). Therefore, our data suggest that stronger inflammation in liver and hyperplasia in CLN observed in infected mice treated with C48/80 may be due to the overproduction of pro-inflammatory cytokines (e.g., IFN-γ and TNF-α) triggered by activated MCs. Our observation is consistent with those in previous reports (Demeure et al., 2005), where a local IgE-independent cutaneous MCs degranulation led to a rapid dermal inflammation and draining lymph nodes hyperplasia. In addition, our data also showed that the mRNA levels of anti-inflammatory cytokine IL-10 in both tissues (CLN and skin), as well as IL-4 in CLN tissue were decreased in the infected mice treated with C48/80 at 9 days p.i., while the levels of IL-4 and IL-10 in both tissues (CLN and skin) were increased in infected mice treated with DSCG at 9 days p.i., respectively. A series of studies had demonstrated that IL4 and IL-10 were regarded as anti-inflammatory cytokines of protective immune responses against fatal Th1-driven pathogenesis in *Pb*ANKA infections (Niikura et al., 2011; Perez-Mazliah and Langhorne, 2015). Therefore, the results from the current study also suggest that the higher mRNA expressions of anti-inflammatory cytokines (e.g., IL-4 and IL-10) in the infected mice treated with DSCG improve pathological process of *Pb*ANKA infection. However, the results presented here may also be due to an indirect effect of released mediators by MCs activation, which leads to production of unmeasured mediators by other cells. Meanwhile, our study did not accurately elucidate whether the examined cytokines (e.g., TNF-α, IFN-γ, IL-4, and IL-10) were released from MCs or other cells.

During *Plasmodium* infection, sequestration of iRBCs resulted in capillary blockage that was prominent in brain, eyes, intestinal villi, and other major tissues and associated with increased BBB leakage, blood retinal barrier leakage, and gastroin-testinal permeability (Wilairatana et al., 1997; Miller et al., 2002; Maude et al., 2014). It was reported that vascular leakage was one of the key events that promoted the pathogenesis of malaria disease (Kim et al., 2011). MCs formed a primary regulator of the integrity and function of the vascular epithelial barrier (Kunder et al., 2011). To evaluate whether MCs activation can promote vascular leakage in response to *Pb*ANKA infection, Evans blue dye staining was performed among different groups in this study. Our data showed that the vascular leakage of liver or lung tissue was notably increased in the infected mice after C48/80 treatment, and whereas notably decreased by the administration of DSCG, demonstrating that MCs activation promoted vascular leakage of liver or lung tissue during *Pb*ANKA infection. Our observation is consistent with the early report showing that the elevated levels of gut mastocytosis had been implicated in impaired gut permeability during *P. yoelii* infection (Potts et al., 2016). Similarly, dengue virus infection can induce MCs-mediated vascular leakage in mice, and the levels of MCs activation were correlated with disease severity in human patients (St John et al., 2013). It was known that activated MCs could promote BBB breakdown in a mouse model of focal cerebral ischemia *via* expression of MMP-9 (McKittrick et al., 2015). In the present study, the levels of MMP-9 in blood, as well as MCs-derived MMP-9 in CLN or skin tissue were significantly elevated in the infected mice with C48/80 treatment, whereas notably decreased in the infected mice with DSCG treatment. Thus, our data suggest that the elevated level of MMP-9 or MCs-derived MMP-9 in mice *via* C48/80 treatment may be correlated with increased vascular leakage of liver or lung tissue during *Pb*ANKA infection.

It is well-known that MCs are the major source of chemokines, which can trigger leukocyte migration to parasite sequestration thereby exacerbating organ-specific inflammation and vascular injury (Ghasemzadeh and Hosseini, 2015; Mukai et al., 2018). The results presented here showed that the total number of leukocyte (including monocytes and neutrophils) in the blood was increased in the infected mice with C48/80 treatment, whereas decreased in the infected mice with DSCG treatment. Our data indicated that MCs activation could induce more leukocytes migration and recruitment during *Pb*ANKA infection in murine model. Previous reports indicated that CCL2 or CXCL1 serves as a powerful attractant of monocytes or neutrophils to inflammatory sites, respectively. To further evaluate the effect of MCs activation on chemokine responses during *Pb*ANKA infection, the levels of CCL2 and CXCL1 in sera, as well as the mRNA expressions of CCR2 (CCL2 receptors) and CXCR2 (CXCL1 receptors) in CLN or liver were assessed among different groups. The results presented here showed that the levels of CCL2 and CXCL1 in sera, as well as CCR2 and CXCR2 in CLN or liver were elevated from the infected mice with C48/80 treatment compared with infected controls at 9 days p.i., whereas reduced from the infected mice by the administration of DSCG. Consistent with our observations, the levels of CCL2 were elevated in the placentae and peripheral blood of women with placental malaria in comparison to healthy control individuals (Ioannidis et al., 2014). Meanwhile, elevated levels of CXCL1 were observed in human endothelial cell lines in response to *P. falciparum*-infected erythrocytes (Tripathi et al., 2009). Given the factor that CCL2 or CXCL1 is secreted by various cell types (e.g., MCs, blood monocytes, alveolar macrophages, fibroblasts, endothelial cells, and epithelial cells) (Mukai et al., 2018), immunofluorescence double-staining for MCs tryptase-CCL2 or -CXCL1 was carried out to confirm whether MCs could release CCL2 or CXCL1 during *Pb*ANKA infection in this study. Our data showed that the increase of CCL2 or CXCL1 released from MCs in CLN or skin was observed from the infected mice with C48/80 treatment compared with the infected control mice at 9 days p.i., while the decline of CCL2 or CXCL1 released from MCs was detected from the infected mice with DSCG treatment. Similarly, blockade of MCs with DSCG had reduced levels of CXCL1 in mice (Chen et al., 2014). Previous reports indicated that MCs-derived CXCL1 can trigger neutrophil recruitment to the site of inflammation (De Filippo et al., 2013; Wezel et al., 2015). MCs-derived CCL2 could result in the elevated levels of CCL2 observed in animal model of Parkinson's disease (Kempuraj et al., 2016). Additionally, CCL2 can recruit monocytes at the site of inflammation in the brain and induce BBB damage (Stamatovic et al., 2005; Yao and Tsirka, 2014), as well as increases endothelial permeability *in vitro* (Stamatovic et al., 2003; Song and Pachter, 2004). Further, other report indicated that depletion of CCR2 prevented CCL2-induced endothelial permeability *in vitro* (Stamatovic et al., 2003). Thus, our data suggest that increase of CCL2 or CXCL1 released from MCs in CLN or skin could contribute to the partial elevated levels of CCL2 or CXCL1 in sera observed in C48/80-induced infected mice at 9 days p.i., which could lead to the enhancement of leukocyte trafficking/infiltration as well as vascular leakage in response to *Pb*ANKA infection. Although it was report that no significant difference in parasitemia was detected from between MC-deficient mice and their wild type controls after *P. yoellii* infection, suggesting that MCs may not be associated with *P. yoelii* parasitemia (Potts et al., 2016). However, MCs-derived TNF triggered by malarial peroxiredoxin decreased *Pb*ANKA parasitemia in mice (Furuta et al., 2006). On the contrary, the number of MCs and the degree of degranulation were correlated with *P. falciparum* parasitemia in patients (Wilainam et al., 2015). Further, other report showed that rising *P. yoellii* parasitemia was related to increased intestinal permeability in mice (Chau et al., 2013). Thus, the higher *Pb*ANKA parasitemia in infected mice via C48/80 treatment may be correlated with the increased vascular leakage during *Pb*ANKA infection. However, other unmeasured chemokines released from MCs may also participate in the process of leukocyte trafficking/infiltration, vascular leakage, as well as parasitemia during *Pb*ANKA infection.

## Conclusion

Collectively, the present data showed that MCs-degranulating agent (C48/80) treatment of *Pb*ANKA-infected mice shorten host survival time, increased parasitemia, and promoted tissue pathology, whereas MCs-stabilizing agent (DSCG) were able to extend survival time, decrease parasitemia, and improve the pathology in infected mice. Although the data related to the MCs depleted or KO mice infected with *Pb*ANKA will be need to collected potentially in future work, the present data indicate that MCs may contribute to pathogenesis of *Pb*ANKA infection through production and secretion of mediators such as cytokines, chemokines, matrix metalloproteinases, enhancement of leukocyte trafficking/infiltration and vascular leakage in this experimental model. Our findings also suggested that selective inhibition of MCs may serve as a potential measure treatment of malaria infection.

## Data Availability

All datasets generated for this study are included in the manuscript/supplementary files.

## Ethics Statement

The animal study was reviewed and approved by the Animal Ethics Committee of Guangzhou University of Chinese Medicine (No. 2012L0816).

## Author Contributions

BH, ZL, and XJ designed and supervised the experiments. BH, SH, XC, XBL, QW, YW, and XL performed the experimental work. BH, SC, RL, ZL, and XJ analyzed the data and wrote the manuscript. BH, KL, HG, SC, RL, ZL, and XJ revised the manuscript.

### Conflict of Interest Statement

The authors declare that the research was conducted in the absence of any commercial or financial relationships that could be construed as a potential conflict of interest. The reviewer LY declared a shared affiliation, with no collaboration, with several of the authors, ZL and RL, to the handling editor at time of review.
